# Prevalence and characteristics of ST131 clone among unselected clinical *Escherichia coli* in a Chinese university hospital

**DOI:** 10.1186/s13756-017-0274-0

**Published:** 2017-11-15

**Authors:** Bin Li, Yanfang Lu, Fangjun Lan, Qingwen He, Chen Li, Yingping Cao

**Affiliations:** 1 Department of Clinical Laboratory, Fujian Medical University Union Hospital, 29 Xinquan Rd, Fuzhou, Fujian 350001 People’s Republic of China; 2 Department of Clinical Laboratory, Maternal and Children’s Health Hospital of Fujian Province, Fuzhou, Fujian 350001 China; 3 Department of Infectious Disease, Fujian Medical University Union Hospital, Fuzhou, Fujian 350001 China

**Keywords:** *E. coli*, ST131, Phylogenetic group, O25*b*, O16, *H*30

## Abstract

**Background:**

*Escherichia coli* clinical sequence type 131 (ST131) has emerged as an extensively antimicrobial resistant *E. coli* clonal group in recent years throughout the world. The aim of this study was to investigate the prevalence and molecular characteristics of ST131 among unselected *E. coli* clinical isolates in a Chinese university hospital.

**Methods:**

Seven hundred consecutive *E. coli* isolates were collected at a Chinese university hospital between 2014 and 2015. Isolates belonging to ST131 were identified by PCR and multilocus sequence typing (MLST), and then characterized for antibiotic resistance, CTX-M-type extended-spectrum β-lactamase genes, fluoroquinolone resistance genes, O types, phylogenetic groups, virulence factors and PFGE patterns.

**Results:**

Overall, 83 (11.6%) isolates were identified as ST131 group. The *H*30 lineage accounted for 53 (63.9%) of the ST131 isolates, including 13 *H*30-Rx and 40 *H*30 non-Rx. The remaining 30 isolates belonged to *H41* lineage. Two O types were identified in this study: O25b (66.3%) and O16 (33.7%). Compared with O25b-B2-ST131 isolates, O16-B2-ST131 isolates harbored less virulence factors of adhesins. ST131 *H*30 Rx isolates had significantly higher virulence score than those of other isolates. O16-B2-ST131 isolates were shown to have a lower resistance to quinolones than O25b-B2-ST131 isolates. 5 nonsynonymous mutations (GyrA S83 L, D87N, ParC S80I, E84V and ParE I529L) were strongly associated with ST131 *H30* and O25b isolates. Results of PFGE demonstrated that these isolates were classified into 68 pulsotypes and these subtypes were grouped into 23 different PFGE clusters using 70% similarity cut-off value.

**Conclusions:**

This is the first study to reveal the prevalence and molecular characteristic of ST131 clonal group among consecutive clinical *E. coli* isolates in China. Our findings demonstrated that ST131 lineage accounts for a small proportion of clinical *E. coli* isolates in China, which included two major groups: O25b-B2-ST131 and O16-B2-ST131. Our results implies that O16-B2-ST131 subclone may be an important type of *E. coli* ST 131 in China, which suggests that future studies should not ignore such clone in this country.

## Background

*Escherichia coli* sequence type 131 (*E. coli* ST131) was identified among extended-spectrum *β*-lactamases (ESBL)-producing isolates in Asia, Europe and North America in 2008, and has rapidly emerged globally to become an important pathogen causing various infections in humans [1, 2]. *E. coli* ST131 is becoming the predominant extraintestinal pathogenic *E. coli* clone which drives multidrug resistance [1].

Although some of *E. coli* ST131 isolates are nontypeable for O antigen, most of these strains are of serotypes O25b and O16 [1, 2]. All *E. coli* ST131 isolates contain the *fimH* gene with high level of allelic diversity and *fimH*30 is the most common one [1]. *H*30 lineage of ST131 is named after *fimH*30 allele and includes two important sublineages (*H*30-R and *H*30-Rx) [1–3]. *E. coli* ST131 isolates are often resistant to fluoroquinolones and produce ESBL [4–6]. The *H*30 subclone comprises most of the fluoroquinolone-resistant ST131 isolates [4]. The main mechanism of fluoroquinolones resistance in *E. coli* ST131 isolates is aminoacid substitutions in the quinolone-resistance determining region (QRDR) of *gyrA*, *parC* and *parE* [1, 2, 6]. Within the *H*30 subclone, the *H*30-Rx subset is strongly associated with CTX-M β-lactamases [1, 2].

Regarding the spread of *E. coli* ST131, most investigations have focused on antimicrobial-resistant isolates, especially those resistant to fluoroquinolones and cephalosporins [1, 2]. *E. coli* ST131 has been detected among extended-spectrum *β*-lactamase-producing or fluoroquinolone-resistant isolates in China [7–9]. However, the prevalence of ST131 among unselected *E. coli* isolates remains unknown. The aim of this study was to investigate the prevalence and molecular characteristics of ST131 in a collection of 700 unselected clinical *E. coli* isolates collected at a university hospital in Southern China.

## Methods

### Bacterial isolates and screening for ST131

A total of 700 non-duplicate *E. coli* clinical isolates were consecutively collected at a Chinese university hospital (Fujian Medical University Union Hospital, Fuzhou, Fujian province, China) between August 2014 and August 2015. These isolates were obtained from urine (51.0%), blood (14.7%) and miscellaneous sources (34.3%). Isolates were identified by the Vitek-2 system GNI card (BioMèrieux, Missouri, France). All isolates were screened for ST131 by PCR-based method for ST131-associated SNPs in *mdh* and *gyrB* as previous described [10]. All the non-O25b, non-O16 and non-phylogenetic group B2 ST131 strains would be confirmed by multilocus sequence typing (MLST) to be ST131 according to the Achtman scheme using seven housekeeping genes (*adk*, *fumC*, *gyrB*, *icd*, *mdh*, *purA* and *recA*) (http://mlst.ucc.ie/mlst/dbs/Ecoli). All ST131 isolates detected were further tested by PCR using specific primers for the *fimH*30 allele for identification of the *H*30 subclone [11]. The *H*30-Rx subclone was identified using allele-specific PCR as previously described [11]. All the *H30*-PCR negative isolates underwent direct sequenceing of *fimH* as previously described [3].

### Susceptibility testing

Antimicrobial susceptibility of *E. coli* ST131 isolates was determined by disk diffusion [12]. Drugs tested included aztreonam (ATM), cefotaxime (CTX), ceftazidime (CAZ), cefepime (FEP), ertapenem (ETP), imipenem (IPM), piperacillin-tazobactam (TZP), ciprofloxacin (CIP), levofloxacin (LEV), amikacin (AMK), and trimethoprim-sulfamethoxazole (SXT). The results were interpreted according to the breakpoints of the 2016 CLSI criteria [12]. *E. coli* ATCC 25922 was used for routine quality control.

### Molecular characterization of ST131

Phylogenetic groups were determined using PCR method described by Clermont et al. previously [13]. Molecular O types were performed on all ST131 isolates by PCR with primers as described previously [14]. The ST131-associated O25b *rfb* variant was detected by a separated PCR assay [15]. The presence of 26 virulence factors (VFs) genes was evaluated by a multiplex PCR method [16].

The virulence score (VF score) was calculated for each isolate as the sum of all virulence-associated genes detected in this study [17]. The sum of all the VF scores of the isolates was then calculated, and finally this sum was divided by the number of isolates to give the mean VF score. *Pap* and *sfa*-*foc* were counted only once regardless of the number of elements or subunits identified [17].

### Detection of *bla*_CTX-M_ gene and fluoroquinolone resistance genes

Isolates nonsusceptible to cefotaxime or ceftazidime underwent detection of *bla*_CTX-M_ using PCR [18]. As to CIP-resistant isolates, mutations in quinolone-resistance determining region (QRDR) of *gyrA*, *parC* and *parE* were determined by PCR and sequencing [19]. Regarding CIP-nonsusceptible or *bla*_CTX-M_ producing isolates, the presence of plasmid mediated quinolone resistance determinants (PMQRs; *qnrA*, *qnrB*, *qnrC*, *qnrD*, *qnrS*, and *aac(6′)-Ib-cr*) were detected by PCR as previously described [20, 21].

### Pulsed-field gel electrophoresis analysis (PFGE) and dendrogram construction

ST131 isolates were subjected to PFGE analysis using XbaI digestion [18]. A PFGE dendrogram was constructed with BioNumerics software (Applied Maths, Sint-Martens-Latem, Belgium) according to the unweighted pair group method based on Dice coefficients. Isolates with a Dice similarity index ≥70% were considered to belong to the same PFGE cluster.

### Statistical analysis

Data were analyzed using the SPSS 19.0. Comparisons of proportions were performed using χ^2^ or Fisher’s exact test (two tailed) or Mann-Whitney test. For each comparison, *p* < 0.05 was considered to be statistically significant.

## Results

### Prevalence of ST131 and its subclones

In total, 83 (11.6%) of the 700 clinical *E. coli* isolates were identified as ST131. ST131 accounted for 15.4% (55/357) of the urinary *E. coli* isolates and 11.7% (12/103) of the bloodstream isolates. *E. coli* ST131 strains were isolated from patients from 23 different wards. Females accounted for 60.2% (50/83) of the ST1193 isolates, and the median age was 62 years (range, 5 years to 89 years).

The results of O typing revealed that ST131 isolates belonged to two types (O25b and O16). All the 83 ST131 isolates were O-antigen typeable. O25b (66.3%) was the most prevalent type, followed by O16 (33.7%) (Table 1). The majority (52/55, 94.5%) of O25b ST131 isolates belonged to *fimH30*, while 96.4% (27/28) of O16 ST131 isolates were *fimH41*subclone. The distribution of O types was not significantly associated with specimen type or source (Table 2).

**Table 1:** Characteristics of *E. coli* ST131 isolates by *fimH* subtyping

Characteristics		ST131 subclones, number of isolates (%)			*P* ^a^		
	Total	H30Rx	H30 non-Rx	H41	H41 vs H30 non -Rx	H41 vs H30-Rx	H30 Rx vs H30 non-Rx
	*N* = 83 (%)	*n* = 13	*n* = 40	*n* = 30			
PCR O type							
O25b	55(66.3%)	12(92.3%)	40(100%)	3(10.0%)	< 0.001	< 0.001	
O16	28(33.7%)	1(7.7%)	0	27(90.0%)			
Antimicrobial resistance							
ATM	30(36.1%)	12(92.3%)	11(27.5%)	7(23.3%)		< 0.001	< 0.001
CAZ	22(26.5%)	10(76.9%)	5(12.5%)	7(23.3%)		0.002	< 0.001
CTX	59(71.1%)	12(92.3%)	28(70%)	19(63.3%)			
FEP	26(31.3%)	11(84.6%)	10(25.0%)	5(16.7%)		< 0.001	< 0.001
TZP	1(1.2%)	0	0	1(3.3%)			
CIP	58(69.9%)	13(100%)	40(100%)	5(16.7%)	< 0.001	< 0.001	
LEV	58(69.9%)	13(100%)	40(100%)	5(16.7%)	< 0.001	< 0.001	
AMK	7(8.4%)	3(23.1%)	1(2.50%)	3(10.0%)			0.042
SXT	56(67.5%)	10(76.9%)	24(60.0%)	22(73.3%)			
*bla*_CTX-M_ type							
CTX-M-14	37(44.6%)	0	22(55.0%)	15(50.0%)		0.001	< 0.001
CTX-M-15	22(26.5%)	12(92.3%)	7(17.5%)	3(10.0%)		< 0.001	< 0.001
CTX-M-14,15	2(2.40%)	0	2(5.0%)	0			
CTX-M-123	1(1.2%)	0	0	1(3.3%)			
No ESBL^b^	21(25.3%)	1(7.7%)	11(27.5%)	9(30.0%)			
FQ^R^ genes^c^							
5 *gyrA*, *parC and parE* mutations^d^	51(61.4%)	13(100%)	35(87.5%)	3(10.0%)	< 0.001	< 0.001	
*aac(6′)-Ib-cr*	14(16.9%)	10(76.9%)	0	4(13.3%)	0.012	< 0.001	< 0.001
*qnr*	4(4.8%)	0	2(5.0%)	2(6.70%)			
Adhesins							
*papAH*	22 (26.5%)	12(92.3%)	6 (15.0%)	4(13.3%)		< 0.001	< 0.001
*papC*	23 (27.7%)	13(100%)	6 (15.0%)	4(13.3%)		< 0.001	< 0.001
*papEF*	22 (26.5%)	12(92.3%)	7(17.5%)	3(10.0%)		< 0.001	< 0.001
*papG allele I*	0	0	0	0			
*papG allele II*	21 (25.3%)	12(92.3%)	6 (15.0%)	3(10.0%)		< 0.001	< 0.001
*sfa/focDE*	1 (1.2%)	0	1(2.50%)	0			
*focG*	0	0	0	0			
*afa/draBC*	7 (8.4%)	0	2(5.0%)	5(16.7%)			
*fimH*	82 (98.8%)	13(100%)	39(97.5%)	30(100%)			
*gafD*	0	0	0	0			
*sfaS*	7 (8.4%)	3(23.1%)	3(7.5%)	1(3.3%)			
Toxins							
*hlyA*	14 (16.9%)	11(84.6%)	1(2.50%)	2(6.70%)		< 0.001	< 0.001
*cnf1*	10 (12.0%)	8(61.5%)	1(2.50%)	1(3.3%)		0.002	< 0.001
*cdtB*	0	0	0	0			
*cvaC*	3 (3.6%)	0	0	3(10.0%)	0.042		
Siderophores							
*fyuA*	81 (97.6%)	13(100%)	38(95.0%)	30(100%)			
*iutA*	77 (92.8%)	13(100%)	38(95.0%)	26(86.7%)			
Capsules							
*kpsMT II*	62 (74.7%)	9(69.2%)	33(82.5%)	20(66.7%)			
*kpsMT III*	0	0	0	0			
*kpsMT K1*	10 (12.0%)	2(15.4%)	5(12.5%)	3(10.0%)			
*kpsMT K5*	47 (56.6%)	11(84.6%)	29(72.5%)	7(23.3%)	< 0.001	0.001	
Miscellaneous							
*nfaE*	0	0	0	0			
*rfc*	0	0	0	0			
*malX*	76 (91.6%)	13(100%)	37(92.5%)	26(86.7%)			
*traT*	73 (88.0%)	13(100%)	32(80.0%.)	28(93.3%)			
Virulence scores^e^	7	9	7	6		0.001	0.001

^a^ *P* values, by either χ^2^ or Fisher’s exact test or Mann-Whitney test, are shown where *P* < 0.05

^b^ESBL, CTX-M-type extended-spectrum-β-lactamase genes

^c^FQ^R^, fluoroquinolone resistance

^d^GyrA S83 L, D87N, ParC S80I, E84V, and ParE I529L

^e^median number of virulence factors (range)

**Table 2:** The sample sources and distribution of virulence factors among different O-serotype of *E. coli* ST131 isolates

Characteristics	Clonal groups, number of isolates (%)			*P* ^a^
	All isolates (*n* = 83)	O25b (*n* = 55)	O16 (*n* = 28)	O25b vs O16
Source				
Urine	55 (66.3%)	36 (65.5%)	19 (67.9%)	
Blood	12 (14.5%)	10 (18.2%)	2 (7.1%)	
Other	16 (19.3%)	9 (16.4%)	7 (25.0%)	
Antimicrobial resistance				
ATM	30 (36.1%)	24 (43.6%)	6 (21.4%)	
CAZ	22 (26.5%)	16 (29.1%)	6 (21.4%)	
CTX	59 (71.1%)	41 (74.5%)	18 (64.3%)	
FEP	26 (31.3%)	22 (40.0%)	4 (14.3%)	0.024
TZP	1 (1.2%)	0	1 (3.6%)	
CIP	58 (69.9%)	49 (89.1%)	9 (32.1%)	< 0.001
LEV	58 (69.9%)	49 (89.1%)	9 (32.1%)	< 0.001
AMK	7 (8.4%)	4 (7.3%)	3 (10.7%)	
SXT	56 (67.5%)	36 (65.5%)	20 (71.4%)	
Adhesins				
*papAH*	22 (26.5%)	19 (34.5%)	3 (10.7%)	0.034
*papC*	23 (27.7%)	21 (38.2%)	2 (7.1%)	0.004
*papEF*	22 (26.5%)	19 (34.5%)	3 (10.7%)	0.034
*papG allele I*	0	0	0	
*papG allele II*	21 (25.3%)	18 (32.7%)	3 (10.7%)	0.034
*sfa/focDE*	1 (1.2%)	1 (1.8%)	0	
*focG*	0	0	0	
*afa/draBC*	7 (8.4%)	2 (3.6%)	5 (17.9%)	0.040
*fimH*	82 (98.8%)	54 (98.2%)	28 (100%)	
*gafD*	0	0	0	
*sfaS*	7 (8.4%)	6 (10.9%)	1 (3.6%)	
Toxins				
*hlyA*	14 (16.9%)	11 (20.0%)	3 (10.7%)	
*cnf1*	10 (12.0%)	8 (14.5%)	2 (7.1%)	
*cdtB*	0	0	0	
*cvaC*	3 (3.6%)	1 (1.8%)	2 (7.1%)	
Siderophores				
*fyuA*	81 (97.6%)	53 (96.4%)	28 (100%)	
*iutA*	77 (92.8%)	52 (94.5%)	25 (89.3%)	
Capsules				
*kpsMTII*	62 (74.7%)	43 (78.2%)	19 (67.9%)	
*kpsMTIII*	0	0	0	
*kpsMT K1*	10 (12.0%)	8 (14.5%)	2 (7.1%)	
*kpsMT K5*	47 (56.6%)	40 (72.7%)	7 (25.0%)	< 0.001
Miscellaneous				
*nfaE*	0	0	0	
*rfc*	0	0	0	
*malX*	76 (91.6%)	51 (92.7%)	25 (89.3%)	
*traT*	73 (88.0%)	46 (83.6%)	27 (96.4%)	
Virulence scores^b^	7 (3 to 11)	7 (3 to 11)	6 (4 to 10)	

^a^ *P* values, by either χ^2^ or Fisher’s exact test, are shown where *P* < 0.05

^b^median number of virulence factors (range)

Subclone typing showed that *H*30 lineage comprised 63.9% (53/83) of the ST131 isolates, including 13 (15.7%) *H*30-Rx and 40 (48.2%) *H*30 non-Rx (Table 1). The remaining 30 isolates belonged to *fimH41* subclone. Among ciprofloxacin-resistant ST131 isolates, 84.5% (49/58) belonged to the *H*30 lineage.

### Antimicrobial susceptibility

Among the 83 ST131 isolates, the highest rates of resistance were to CTX (71.7%), CIP (69.9%), LEV (69.9%) and SXT (67.5%). On the contrary, resistance rates were low to ATM (36.1%), FEP (31.3%), CAZ (26.5%), AMK (8.4%) and TZP (1.2%). None of the isolates was resistant to imipenem or ertapenem. These findings are summarized in Table 1.

Compared with O25b-ST131 isolates, O16-ST131 isolates were shown to have a lower resistance to fluoroquinolones (CIP and LEV) and FEP, and had a similar resistance to third generation cephalosporins (CTX and CAZ) (Table 2).

### CTX-M ESBL and fluoroquinolone resistance mechanisms

*bla*_CTX-M_ genes were harbored by 62 (74.7%) *E. coli* ST131 isolates. Of these, 37 isolates carried *bla*_CTX-M-14_, 22 isolates carried *bla*_CTX-M-15_ and 1 isolates carried *bla*_CTX-M-123_. The remaining 2 isolates co-produced *bla*_CTX-M-14_ and *bla*_CTX-M-15_.

All CTX-M-14 producers were *H*30 non-Rx and *H41*, whereas most ST131 *H30*-Rx subclones were CTX-M-15 producers (92.3%, 12/13; Table 1).

All 58 ciprofloxacin-resistant ST131 isolates contained 4 or 5 nonsynonymous mutations in *gyrA*, *parC* and *parE*. All these ST131 isolates had a set of 3 conserved QRDR amino acid substitutions (GyrA S83 L, D87N and ParC S80I). The presence of 5 mutations (GyrA S83 L, D87N, ParC S80I, E84V and ParE I529L) was significantly more prevalent among *H*30 isolates than *H41* (*P* < 0.001). These findings are summarized in Table 1. Meanwhile, these 5 mutations were strongly associated with O25b subclones (Table 3).

**Table 3:** Fluoroquinolone resistance mechanisms, O types and *fimH* subclone among 83 ST131 *E. coli* isolates

CIP^a^	*fim*H type (n)	O type (n)	N	gyrA	parC	parE	aac(6′)-Ib-cr	qnr (n)
R	H30	O25b	48	S83 L, D87N	S80I, E84V	I529L	9	*qnrS1*(1)
R	H41	O16	3	S83 L, D87N	S80I, E84V	I529L	1	*qnrB1*(1)
R	H30	O25b	1	S83 L, D87N	S80I	P415L	–	–
R	H41	O16	1	S83 L, D87N	S80I	I529L	–	–
R	H41	O16	1	S83 L, D87N	S80I	S458A	1	–
R	H41	O16	3	S83 L, D87N	S80I	L445H	1	–
R	H41	O25b	1	S83 L, D87N	S80I	L445H	–	–
I	H41	O25b	1	ND^b^	ND	ND	1	*qnrB1*(1)
S	H41	O16	19	ND	ND	ND	1	–
S	H41	O25b	2	ND	ND	ND	–	*qnrS1*(1)^c^
S	H30	O25b	3	ND	ND	ND	–	–

^a^R, resistant; I, intermediate; S, susceptible

^b^ND, not detected

^c^In this isolate, *bla*_CTX-M-15_ was positive

Three types of PMQR determinants were found in 16 ST131 isolates, including *aac(6′)-Ib-cr* (*n* = 14), *qnrS1* (*n* = 2) and *qnrB1* (n = 2). Two ST131 isolates coproduced *qnrB1* and *aac(6′)-Ib-cr*. *aac(6′)-Ib-cr* were significantly concentrated in *H30*-Rx isolates than non H30-Rx isolates (*P* < 0.001) (Table 1).

### Virulence factors

The most frequent VF genes were *fimH* (type 1 fimbriae), *fyuA* (yersiniabactin), *iutA* (iron uptake gene), *malX* (pathogenicity-associated island marker), *kpsMT II* (group II capsule), and *traT* (outer membrane lipoprotein), each of which was detected in ≥60% of the isolates. In contrast, 8 genes were each identified in less than 10% of isolates, including cvaC, *sfa/focDE*, *sfaS*, *afa/draBC*, *focG*, *papG* allele I, *gafD*, *cdtB*, *kpsMTIII*, *nfaE* and *rfc*. The median virulence score was 7 (range, 3 to 11). Among the two O groups, the median virulence scores (ranges) were 7 (3 to 11) for O25b-ST131 and 6 (4 to 10) for O16-ST131, respectively.

ST131 *H30*-Rx isolates possessed the highest virulence score (mean, 9; range, 7 to11). ST131 *H30* non-Rx and *H41* isolates showed similar virulence scores and ranges of VFs (mean scores, 7 [range, 4 to 9] and 6 [range, 3 to 9], respectively). ST131 *H30*-Rx isolates had significantly higher virulence score than *H30* non-Rx and *H41* isolates (Table 1). Four adhesins (*papAH*, *papC*, *papEF* and *papG allele II*) and two toxins (*hlyA* and *cnf1*) were significantly more frequent among ST131 H30-Rx isolates (Table 1).

### Bacterial clonal relatedness

**Fig. 1:**
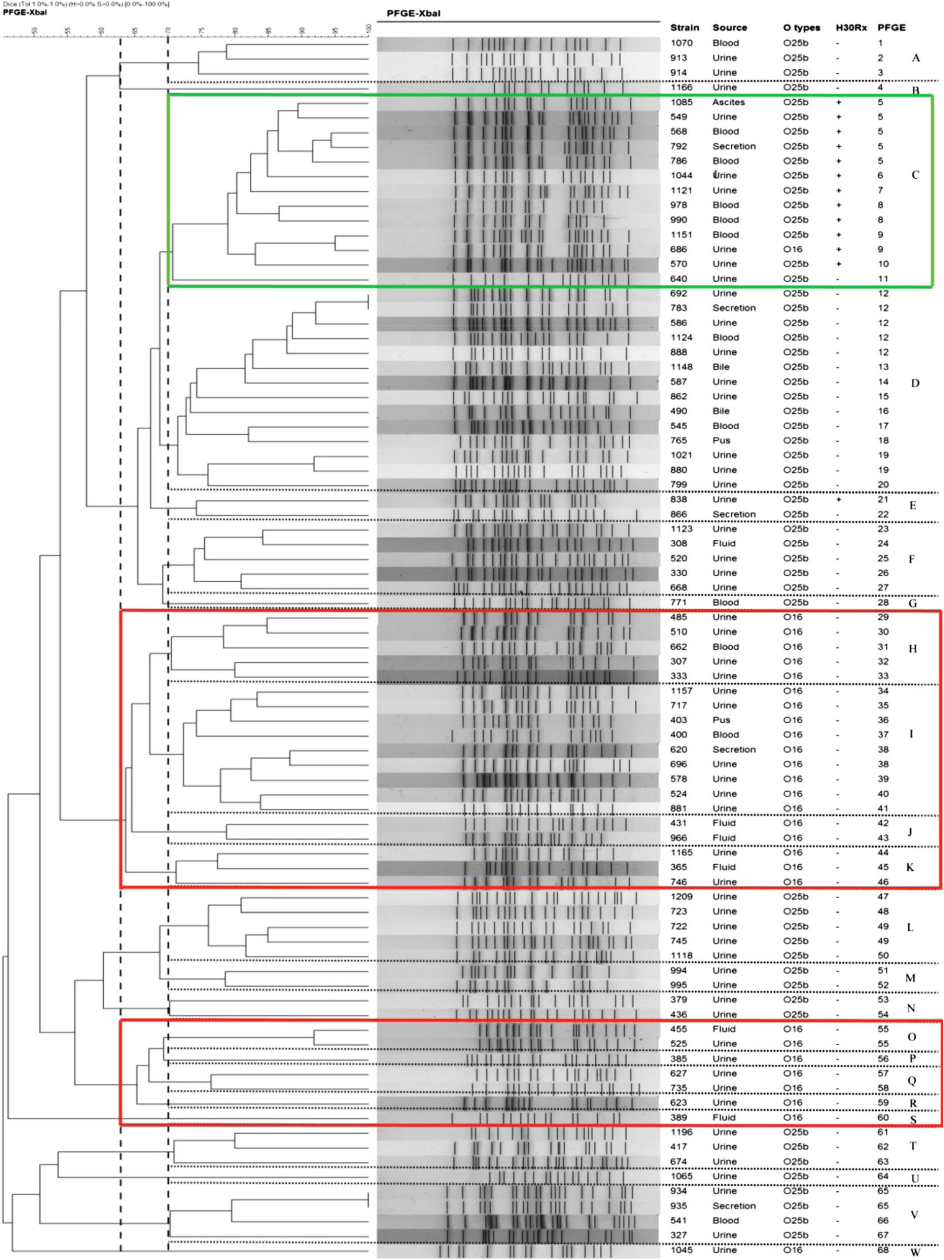
Pulsed-field gel electrophoresis (PFGE) of XbaI-digested DNA from 83 ST131 *E. coli* isolates. Strain designation, Sample source, O serotype, *H*30 Rx and PFGE patterns are shown. Green box indicated the main cluster of ST131 *H*30 Rx isolates using 70% similarity cut-off value. Red box showed the two main clusters of O16 ST131 isolates at 63% similarity level

PFGE analysis of the 83 *E. coli* ST131 isolates demonstrated that these isolates were classified into 68 pulsotypes (named 1–68, Fig. 1) and these subtypes were grouped into 23 different PFGE clusters (named A-W, in Fig. 1) using 70% similarity cut-off value. Two PFGE clusters (C and D) were predominant, grouping 13 and 14 isolates, respectively, whereas the other 21 PFGE clusters contained up to 9 ST131 isolates each (Fig. 1). Cluster C contained 92.3% (12/13) of *H30*-Rx isolates. The majority of O16 ST131 isolates belonged to two clusters (H and I), grouping 5 and 9 isolates. As shown in Fig. 1, at 63% similarity level, O25b-ST131 isolates were clustered into six major clonal groups, while 92.9% of the O16 isolates were clustered into two clonal groups (group one contained clusters H, I, J and K, and group two contained clusters O, P, Q, R and S).

## Discussion

To our knowledge, this is the first molecular survey reporting the prevalence and characteristics of *E. coli* ST131 and its subclones among unselected clinical isolates in China. We found *E. coli* ST131 accounted for 11.6% of all *E. coli* isolates, which is similar to rates reported previously [1, 22, 23]. In addition, there was a similar distribution of ST131 among urine and blood samples. Meanwhile, the PFGE study indicated that ST131 isolates in this study were highly diverse at the pulsotype level and there was no predominated clone (Fig. 1).

O25b was previously considered the predominant ST131 clone type, while O16 isolates accounted for a small percentage [1, 2, 24]. Similar to previous studies [23, 24], O25b-B2-ST131 in this study was the predominant clone among clinical *E. coli* isolates. Notably, a high percentage (33.6%) of O16-B2-ST131 was found in the present study. In China, a recent study found that O16-B2-ST131 accounted for the predominant subset among ST131 fecal *E. coli* isolates [25]. However, among *E. coli* clinical isolates, all other researches focused on O25b-B2-ST131 in our country and no data are available on the prevalence of O16-B2-ST131 among *E. coli* clinical isolates till now, the present study is the first to show the presence of O16-B2-ST131 in this country. Taken together with the previous study [25], these findings suggest that O16-B2-ST131 may emerge as an important type of *E. coli* ST131 in China. The rate of O25b-B2-ST131 was not striking in this study. However, the O16-B2-ST131 may need special attention for the high percentages found in our results and previous study [25]. As discussed before, the clonal group should be investigated in the future study in other geographical regions in China [25, 26].

The *H*30 lineage was a very important ST131 subclone, which firstly appeared in the year of 2000 and expanded rapidly to become the most dominant and extensively distributed multidrug-resistant lineage of *E. coli* worldwide [1, 2]. Within the *H*30 subclone, the *H*30-Rx subset is a major drug-resistant pathogen among fluoroquinolone-resistant *E. coli* isolates and is associated with *bla*_CTX-M-15_ [1, 2]. In this study, we also explored the prevalence of *H*30 lineage and *H*30-Rx sublineage among *E. coli* ST131 isolates. *H*30 lineage accounted for the majority of ST131 isolates in our study, which is similar to those in previous studies [22, 23]. However, the prevalence of *H*30-Rx ST131 was obviously lower than those reported in other researches [23, 27]. Meanwhile, the great majority of *H*30-Rx isolates harbored *bla*_CTX-M-15_ in this study. Resistances to 11 most commonly used antimicrobials were evaluated in this study. Our findings support the strong association of *H*30-Rx sublineage with multidrug resistance and the presence of *bla*_CTX-M-15_ (Table 1). We also identified the *H*30-Rx with the presence of *aac(6′)-Ib-cr* (Table 1). Resistance of fluoroquinolones is mainly due to chromosomal mutations in the QRDR regions, especially in gyrA and parC [28, 29]. In this study, A set of 3 conserved mutations in QRDR regions (GyrA S83 L, D87N, and ParC S80I) could be found in all the CIP-resistant ST131 isolates. These chromosomal mutations have been previously linked to fluoroquinolone resistance [23, 30]. Meanwhile, 5 mutations (GyrA S83 L, D87N, ParC S80I, E84V and ParE I529L) were strongly associated with *H30* ST131 isolates, which is similar to the previous studies [23, 31].

Regarding virulence factors, *fimH*, *fyuA*, *iutA*, *kpsMTII* and *traT* were the most prevalent virulence factors found in this study, which have been associated with *E. coli* ST131 isolates in previous studies [1, 32]. There was a similar prevalence rate for these five virulence factors within different O types ST131 isolates (Table 2). O16-B2-ST131 isolates harbored similar number of virulence genes than O25b-ST131 isolates. Compared with O25b-B2-ST131 isolates, O16-B2-ST131 isolates harbored less virulence factors of adhesins (*papAH*, *papC*, *papEF* and *papG alleleII*).The *pap* adhesion genes probably played an important role in the pathophysiology of pyelonephritis caused by *E. coli* [33, 34]. This property probably suggests that O16-B2-ST131 isolates have a lower adhesive ability and pathogenicity than O25b-B2-ST131. Meanwhile, O25b-B2-ST131 isolates were significantly more likely to possess *kpsMT K5* than O16-B2-ST131 isolates. *kpsMT K5* is a capsule synthesis gene [16]. The possession of the gene may enhance the pathogenesis of O25b-B2-ST131 isolates due to the evasion of phagocytosis (capsule encoded by *kpsMT K5*).

In conclusion, to our knowledge, this is the first report on the prevalence and molecular characteristic of ST131 clonal group among consecutive clinical *E. coli* isolates in China. Our findings demonstrated that ST131 lineage accounts for a small proportion of clinical *E. coli* isolates in China, which included two major groups: O25b-B2-ST131 and O16-B2-ST131. Our results implies that O16-B2-ST131 subclone may be an important type of *E. coli* ST 131 in China, which suggests that future studies should not ignore such clone in this country.

## Abbreviations

*E. coli*: *Escherichia coli*
ESBL: Extended-spectrum *β*-lactamases
MLST: multilocus sequence typing
QRDR: Quinolone-resistance determining region; PFGE**:** Pulsed-field gel electrophoresis analysis
ST: Sequence type
VF: Virulence score
